# Service Life Assessment of Historical Building Envelopes Constructed Using Different Types of Sandstone: A Computational Analysis Based on Experimental Input Data

**DOI:** 10.1155/2014/802509

**Published:** 2014-07-07

**Authors:** Václav Kočí, Jiří Maděra, Jan Fořt, Jaromír Žumár, Milena Pavlíková, Zbyšek Pavlík, Robert Černý

**Affiliations:** Department of Materials Engineering and Chemistry, Faculty of Civil Engineering, Czech Technical University in Prague, Thákurova 7/2077, 166 29 Prague 6, Czech Republic

## Abstract

Service life assessment of three historical building envelopes constructed using different types of sandstone is presented. At first, experimental measurements of material parameters of sandstones are performed to provide the necessary input data for a subsequent computational analysis. In the second step, the moisture and temperature fields across the studied envelopes are calculated for a representative period of time. The computations are performed using dynamic climatic data as the boundary conditions on the exterior side of building envelope. The climatic data for three characteristic localities are experimentally determined by the Czech Hydrometeorological Institute and contain hourly values of temperature, relative humidity, rainfalls, wind velocity and direction, and sun radiation. Using the measured durability properties of the analyzed sandstones and the calculated numbers of freeze/thaw cycles under different climatic conditions, the service life of the investigated building envelopes is assessed. The obtained results show that the climatic conditions can play a very significant role in the service life assessment of historical buildings, even in the conditions of such a small country as the Czech Republic. In addition, the investigations reveal the importance of the material characteristics of sandstones, in particular the hygric properties, on their service life in a structure.

## 1. Introduction

Cultural heritage is comprised of tangible and intangible parts. It can be considered as an image of national advancement, history, or education which should be passed on future generations. Its preservation is therefore highly valued by each nation. Historical monuments and structures represent a major part of tangible cultural heritage. In Europe, they were often made of natural stones, which were the dominant building material until the industrial revolution in the 19th century allowed manufacturing high quality cement for the production of reinforced concrete [1]. Since that time, building stone is used in Europe for its decorative appearance mainly and its application for load bearing structures is limited [2]. Anyway, natural stones are subjected to deterioration processes causing their damage and subsequent reduction of their service life. These processes can be at least decelerated using precise damage diagnosis which can be considered an effective tool for the conservation of natural stones and preservation of historical buildings [1], because it can provide necessary data for a comprehensive characterization or damage prediction or it could be helpful to choose the right techniques and methods to preserve the built heritage. In particular, understanding the hygrothermal behavior of climatically exposed components and structures of historical buildings represents the first step in avoiding damage or the undue heat loss from constructions.

There are several different processes involved in the stone decay [3–5]. However, salt crystallization and freeze/thaw cycles may be identified as the most impacting ones. Salt crystallization is a very complex phenomenon which influences the service life of most buildings. Moreover, the risk of salt induced degradation can be higher in marine environment [6], where the salt concentration is usually higher. Freeze/thaw cycles can play a significant role especially in the cold regions or regions with substantial temperature changes on the daily basis [7]. The decay principles of both phenomena are similar. Salt crystallization as well as water freezing is characterized by a volume increase [3]. Thus, the pressure within the pores is increased until the tensile strength of the stone is reached. At this point, new microfractures are developed or the present ones are widened, which causes deterioration of mechanical properties and thus decrease of the service life. Therefore, many research studies are based on an experimental approach under laboratory conditions aimed at the determination of freeze/thaw or salt crystallization effects on the properties of stones, trying to estimate the remaining service life. Saad et al. [8] analyzed the effect of freeze/thaw cycles on mechanical properties, porosity, and water transport parameters of two sandstones and eight limestones. They tried to find correlation between these two groups of parameters but were unable to classify all the studied rocks. Karaca et al. [9] monitored changes of abrasion values of several types of stones (marble, limestone, travertine, onyx, and granite). They investigated fresh samples and samples exposed to 28 freeze/thaw cycles. However, the presented method of theirs was finally not recommended for onyxes and granites, because they did not respond well to 28 freeze/thaw cycles and thus did not provide meaningful results. Gloss values changes of polished stone surface caused by the effect of freeze/thaw cycles and the correlation between the basic physical properties and mechanical properties were investigated by Ozcelik et al. [10]. They studied four types of carbonate rocks using a thermostatic chamber, gloss meter, and microphotography. Cardell et al. [4] performed chemical, mineralogical, and porosimetric analyses of limestone exposed to the penetration of different salt solutions in order to identify the effect of salt crystallization.

As it is often impossible to obtain samples for laboratory studies due to the necessary architectural heritage protection, another approach for assessing the quality of stones is based on the application of noninvasive techniques, such as the ultrasonic methods reported in [11] or combined geophysical approaches applied in [12]. Computational modeling of coupled heat and moisture transport presents an alternative option in that respect as most decay processes of natural stones are closely related to the heat and moisture transport. A precise knowledge of moisture and temperature fields in the constructions can serve as an excellent ground for service life prediction, as it was demonstrated for other building materials before [13]. However, the crucial factor of this approach is to use as precise and realistic input parameters as possible; otherwise the results can be substantially distorted. That means it is necessary to use dynamic weather data related to the location of analyzed buildings. Furthermore, the principal parameters of studied materials have to be measured in an extensive way. Experimental measurements of material properties should cover transport and storage parameters of heat, water, and water vapor, as well as basic physical properties. Additionally, all the parameters, if possible, should be measured as a function of moisture content, because it can affect the properties of building materials in a significant way [14, 15]. Because of the limited transport possibilities in the past, historical buildings were usually made of materials found in the nearest locations. There were several exceptions, of course, and some historical buildings were made of stones from more distant quarries, but most of historical buildings meet this rule. Therefore, the stone samples for determination of material parameters do not need to be taken in situ; they can be obtained from regional quarries with similar mineralogical composition. This fact makes it possible to classify this approach as noninvasive, which suits well to the overall heritage protection. However, it is important to realize that also properties of two samples within the same quarry can be different due to lateral and vertical facies variations, so it is impossible to obtain stone samples with identical properties as the stones used in the past. However, experimental analysis performed on a large number of samples can at least minimize this negative effect.

In this paper, we focus on the investigation of hygro-thermo-mechanical performance of three different kinds of sandstones that are presently applied in the renovation of historical monuments in the Czech Republic. Since most old quarries that were used for the extraction of building stones in the past are presently not active, a particular attention should be paid to the application of stones whose properties are as close as possible to the original materials. In that respect, detailed preliminary considerations taking into account the material color, structure, general appearance, and mechanical and other physical properties are supposed to be done, in order to find compatible materials with historically applied stones. One should also take into account the durability properties of building stones, that is, their resistance to the harmful climatic effects. In the past centuries, a plaster or another finishing layer was often missing and the stones formed the external parts of historical buildings and bridges. Therefore, the laboratory measurement of basic physical, mechanical, hygric, thermal properties and durability properties of the analyzed building stones is done, in order to get reliable input data for the subsequent computational modeling of their performance under real climatic conditions corresponding to several different Czech regions.

## 2. Materials and Methods

### 2.1. Studied Materials

**Figure 1:**
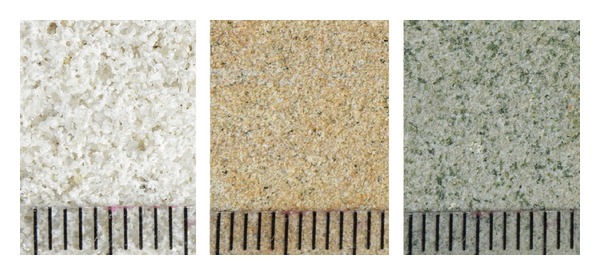
Images of investigated sandstones (SK-SL-SZ).

**Figure 2:**
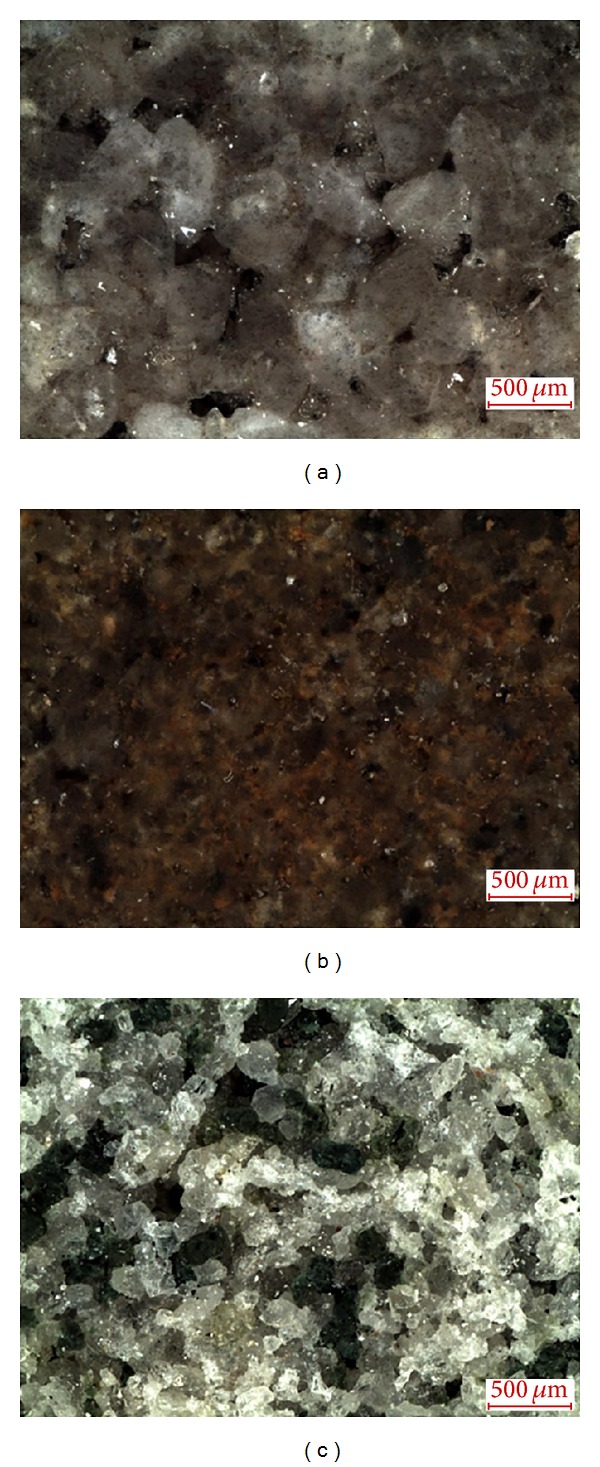
Microscopy images of investigated sandstones (SK—(a), SL—(b), and SZ—(c)).

Three different types of sandstone originating from the quarries in the Czech Republic were analyzed. The particular materials are denoted as SK, SL, and SZ; the quarries locations, age, petrography, and mineralogical composition are given in Table 1. The pictures of sandstones, including microscopy images, are shown in Figures 1 and 2.

The chemical composition of studied sandstones was determined by X-ray fluorescence (XRF) analysis.  Table 2 shows that the main component of all of them was silica; the differences were mainly in the amounts of alumina and ferric oxide which, however, did not exceed 5%.

### 2.2. Experimental Methods

#### 2.2.1. Basic Physical Characteristics

The bulk density, matrix density, and total open porosity were investigated as the basic physical characteristics. Bulk density was measured on the gravimetric principle, using the sample size measured by a digital length meter and its dry mass. For this measurement, 10 cubic samples of side 100 mm were used. The matrix density was determined by helium pycnometry using Pycnomatic ATC (Thermo). At the application of Pycnomatic ATC, a well dried sample of studied material is weighed and placed in a calibrated reference chamber of known volume. Helium is first loaded at known pressure in a calibrated reference chamber and then expanded into the sample chamber. Once the pressure is stabilized, experimental data are collected and the material volume is accessed. The accuracy of the gas volume measurement using this device is ±0.01% from the measured value, whereas the accuracy of used analytical balances is ±0.0001 g. On the basis of bulk density and matrix density measurements, the total open porosity was calculated in a common way [16]. The relative expanded uncertainty of the applied testing method was 5%.

#### 2.2.2. Mechanical Properties

Mechanical properties of researched stones were characterized by compressive strength and Young's modulus. The compressive strength was measured on cubic samples of 50 mm side using the standard procedure described in [17].

Young's modulus was determined using the pulse ultrasonic method and calculated according to

(1) $$\begin{array}{c} E_{D}=\frac{m}{abl}{\left(\frac{c}{t}\right)}^{2}, \end{array}$$

where *m* is the mass of the specimen, *l* the length, *a* the height, *b* the width of the specimen, *t* the transmission time of the signal through the specimen in the direction *l*, respectively, and *a* or *b* and *c* one of the dimensions *l*, *a*, or *b*, depending on the direction of measurement. In the experiments, the prismatic samples had the dimensions of 40 × 40 × 160 mm; the measurements were performed in longitudinal direction using a DIO 562 device working on the frequency of 50 kHz.

#### 2.2.3. Durability Properties

Freeze/thaw resistance was measured for the investigated materials. It was calculated as a ratio of compressive strength of the frost loaded material and reference material without any freezing/thawing cycles exposure. According to the European standard [18], a single freeze/thaw cycle required 6 hours freezing at −15°C and 6 hours thawing in 20°C warm water. The total number of applied cycles was 70.

#### 2.2.4. Water Transport Properties

Transport of water in liquid phase was described by moisture diffusivity which was determined as a function of moisture content. For that purpose measurement of moisture profiles was done, using a vertical experimental setup. In the experiment, rod-shaped samples with the dimensions of 20 × 40 × 290 mm were used. Epoxy resin was employed for water- and vapor-proof insulation on the lateral sides to assure 1D moisture transport. In the determination of moisture profiles, the specimens were put in contact with water, whereas the initial state was dry material. After choosing time intervals, the samples were cut into several pieces. The moisture content was then determined in each piece by the gravimetric method. Duration of the suction experiment was 30, 60, and 90 minutes. For the determination of moisture diffusivity, an inverse analysis of experimentally measured moisture profiles was used [19].

#### 2.2.5. Water Vapor Transport Properties

Water vapor transport was characterized by water vapor diffusion permeability *δ* (s), diffusion coefficient of water vapor *D* (m^2^/s), and water vapor diffusion resistance factor *μ* (—). The measurement of water vapor transport parameters was carried out under isothermal conditions using the steady-state cup method according to the European standard [20]. It is based on simulating one-dimensional water vapor diffusion and measuring the diffusion water vapor flux through the specimen and partial water vapor pressure in the air under and above specific specimen surface. Water vapor transport properties of building materials are generally considered as depending on the relative humidity. On this account, two relative humidity pairs were applied in the cup measurements, namely, 0/50% (dry cup) and 98/50% (wet cup).

#### 2.2.6. Water Vapor Storage Parameters

The sorption and desorption isotherms were measured using a DVS-Advantage device (Surface Measurement Systems Ltd.). The instrument measures the uptake and loss of vapor gravimetrically, using highly precise balances having a resolution of 10 *μ*g [21]. The humidity range of the instrument is 0–98% with the accuracy ±0.5%. Values of the relative humidity are measured by the dew point calculations, using an optical sensor, that is, by knowing the saturated vapor pressure at a given temperature under the atmospheric pressure. Before the measurements, samples of the studied materials were dried at 105°C. After the drying, they were put into the climatic chamber of the DVS-Advantage instrument and hung on the automatic balances in a special steel tube. The experiments were performed at 20°C. The samples were exposed to the following partial pressure profile: 0, 20, 40, 60, 80, and 98% relative humidity. During the experiments, the DVS-Advantage instrument was running in the *dm*/*dt* mode (the mass variation over the time variation) to decide when the equilibrium was reached. A fixed *dm*/*dt* value of 0.00004 g/min was selected for all relative humidity segments.

#### 2.2.7. Heat Transport and Storage Parameters

Thermal conductivity *λ* (W/mK) and volumetric heat capacity *c*_*v*_ (J/m^3^K) were measured using an ISOMET 2114 device (Applied Precision Ltd.). The measurement is based on an analysis of the temperature response of the analyzed material to heat flow impulses. The heat flow is induced by electrical heating using a resistor heater having a direct thermal contact with the surface of the sample. The reproducibility of ISOMET 2114 for thermal conductivity measurement is 3% of reading + 0.001 W/mK and for volumetric heat capacity 3% of reading + 1 · 10^3^ J/m^3^K. The measurement accuracy is given in Table 3.

The experiments were carried out for both dry and water fully saturated materials at the standard laboratory temperature of 20°C. The particular cubic samples with a side dimension of 150 mm were measured using a surface probe. During the experiment, temperature of probes changed dynamically between approximately 20 and 35°C.

### 2.3. Numerical Simulation

#### 2.3.1. Mathematical Model and Computer Simulation Tool

The moisture and heat transport was described by the balance equations formulated in Künzel's mathematical model [22]. According to the description of water transport, this model can be classified as diffusion type. It is probably the most frequently used mathematical model in present days. One of the advantages of this model is that for the description of coupled water and water vapor transport the relative humidity of air *φ* is used. Thanks to that, moisture transport in multilayered constructions can be expressed by a continuous quantity on the material interfaces. Künzel's mathematical model also contains several simplifications while the main phenomena are kept. For instance, in the balance equations of heat and moisture there is an assumption that change of amount of gaseous moisture in time can be neglected. Also the latent heat of water freezing is neglected. Because of its simplicity and accessibility of relatively tight set of input characteristics this model became favorite for many investigators. It has been verified and successfully applied in numerous hygrothermal simulations before; see for example [23].

The calculations were performed using the computer code HMS Transport 1.0 [24], which utilizes the general computer simulation tool SIFEL [25] based on the finite-element method. HMS was developed at Department of Materials Engineering and Chemistry, Faculty of Civil Engineering, Czech Technical University in Prague. Because of its user friendly graphical interface, it easily allows setting up all the necessary requisites, such as the problem type (1D, 2D, or 3D), mesh construction, material and boundary conditions assignment, mathematical model selection, or time specification of computations. Although this code allows preprocessing of up to three-dimensional problems, in this paper it was used only for a one-dimensional problem, because this type of heat and moisture transport between the interior and exterior through the analyzed wall was the most essential type in this case. Two- or three-dimensional modeling is mostly used for the simulation of the behavior of whole constructions or at least their complex parts only [26, 27].

#### 2.3.2. Description of the Building Envelope

**Figure 3:**
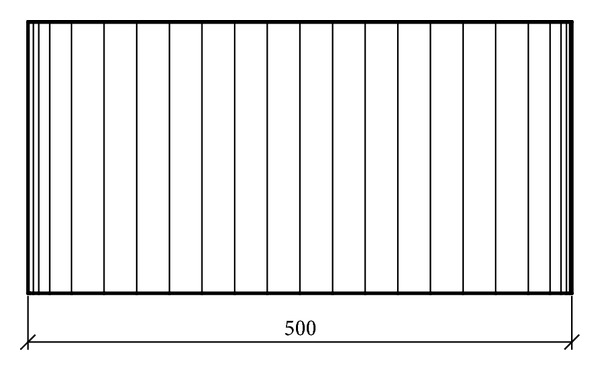
Finite-element scheme of the analyzed external wall.

A natural stone-based external wall without any external and internal finishes was analyzed. This corresponded to the historical masonry habits, which preferred natural appearance of stones. The thickness of the wall was assumed to be 500 mm. At the solution by the finite-element method, the wall was divided into several segments according to the scheme depicted in Figure 3.

#### 2.3.3. Boundary Conditions

Dynamic climatic data in the form of test reference years obtained from three meteorological stations in different locations of the Czech Republic were used. These stations are operated by the Czech Hydrometeorological Institute providing official weather data for the Czech Republic. All the weather parameters are gathered every hour for at least thirty years. Only such extensive database can serve as a background for creation of reference year. The data involve long-term hourly average values of temperature, relative humidity, amount of rainfall, wind velocity and direction, and several kinds of sun radiation.

Prague, as one of these locations, represents the capital city of the Czech Republic. It is located approximately in the geographic center of the country and has the average altitude of 250 meters. Prague's climate is mild with the highest average temperature of 17.6°C in August. The coldest months are December, January, and February with average temperatures between −2.2 and −3.6°C. Annual average amount of rainfalls is 526 mm.

The second location is Holešov in the southeastern part of the Czech Republic. This region is ranked as the warmest region in the country, with the highest average temperature of 19.5°C in August. In winter, only January's average temperature drops below zero, reaching −2.6°C.

**Figure 4:**
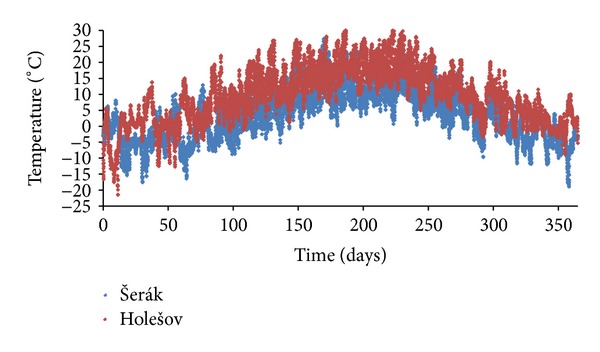
A comparison of reference-year temperatures for Šerák and Holešov.

The third locality, Šerák, represents the harshest climatic conditions in the Czech Republic. The meteorological station is located at 1328 meters above the sea level in the mountains of the northeastern part of the country. In a comparison with Holešov, the annual average temperature is almost 6°C lower, while the average relative humidity is about 5% (absolute) higher. A comparison of the temperatures in Šerák and Holešov over a reference year is shown in Figure 4.

On the interior side of the building envelopes, constant values of temperature 21°C and relative humidity 55% were applied. These values are prescribed in the thermal standard [28].

### 2.4. Description of the Service Life Assessment Process

**Figure 5:**
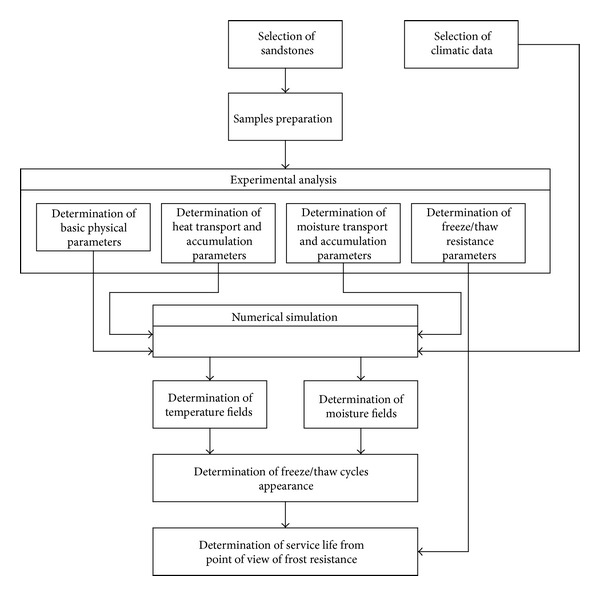
Flowchart of the service life assessment process.

Central Europe belongs to the geographical areas which are characterized by an alternation of the freezing and thawing periods. In such climatic conditions, a typical damage caused by the external environment is due to the freeze/thaw cycles in the external surface layers of building structures [29]. Therefore, also in this paper the service life of investigated building envelopes was assessed primarily from the point of view of freeze/thaw resistance. In the assessment process a combination of experimental analysis and numerical simulation was used. Its flowchart is presented in Figure 5.

## 3. Results and Discussion

### 3.1. Experimental Measurements

The basic physical parameters of investigated materials are given in Table 4. The highest open porosity had the sandstone SZ from the locality Záměl and the lowest SK from Kocbeře.

Mechanical properties of sandstones are summarized in Table 5. Presented values of compressive strength and Young's modulus depend on many factors such as grain size, anisotropy, or pore size distribution of sandstones. Comparing the values of mechanical properties with total open porosity presented in Table 4, certain relationship can be observed. However, the mineralogical composition could play an important role as well because the sandstone SL from the locality Libnava had the highest compressive strength and Young's modulus although it had slightly higher porosity than SK.

The freeze/thaw resistance (Table 6) of all studied sandstones was relatively high. The best performance exhibited the sandstone SL with the highest values of compressive strength and Young's modulus (Table 5); the worst showed SK with the lowest open porosity (Table 4). This absence of any clear correlation to the mechanical properties and porosity can be attributed to the natural character of the studied materials and their inhomogeneity.

**Figure 6:**
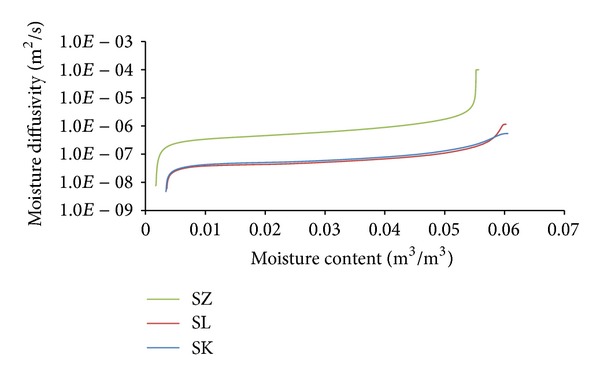
Moisture diffusivity versus moisture content functions.

Moisture diffusivity  *κ* as a function of moisture is presented in Figure 6. In this case, the obtained results were in a good qualitative agreement with the open porosity (Table 4). The highest  *κ* in the whole range of moisture content exhibited the sandstone SZ with the highest porosity; the  *κ* values of the materials SK and SL were similar, in particular taking into account the measurement accuracy.

The measured water vapor transport properties are summarized in Tables 7 and 8. The water vapor diffusion resistance factors were similar for all three analyzed sandstones and relatively low as compared with the most common building materials. The high diffusion permeability of the sandstones is certainly a good feature from a point of view of their durability; a fast removal of water vapor may prevent a structure from water condensation and accumulation inside.

**Figure 7:**
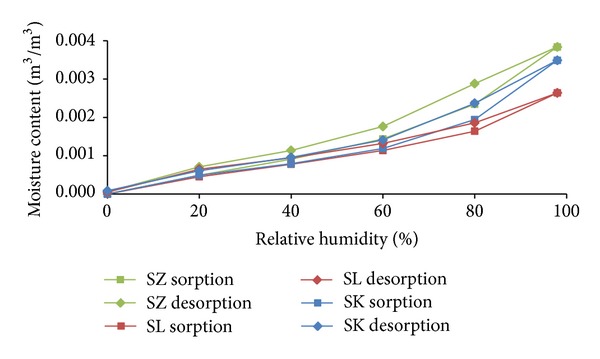
Sorption and desorption isotherms.

Sorption and desorption isotherms of studied sandstones are given in Figure 7. Apparently, the water vapor accumulation was low for all sandstones, as it could be realized in the building materials only.

The results of the measurement of thermal parameters are given in Table 9. The thermal conductivity was in a qualitative agreement with the open porosity (Table 4); SZ with the highest porosity had the lowest thermal conductivity. The differences in the volumetric heat capacity were low; they reflected only the differences in the bulk density of the investigated sandstones (Table 4). The presence of moisture was important for all three sandstones and affected both thermal conductivity and volumetric heat capacity in a significant way.

### 3.2. Numerical Simulations

As the hygric and thermal properties of building materials used as input parameters of the computational model depend on the state variables such as temperature or moisture content, their values in the particular positions in the building envelope can change gradually, based on cyclic fluctuations of moisture and temperature. Therefore, the results of hygrothermal simulations have to be evaluated after certain period of time which is necessary for the properties to achieve a kind of dynamic equilibrium in a sense that the properties used during one test reference year are very close to those used for the subsequent year. According to the previous experience, the time period of five years is mostly long enough for a development of cyclic hygrothermal behavior of building envelopes which is not established yet in the first years of simulation. Therefore, the results of hygrothermal performance of natural stone-based building envelopes presented in this paper will be related to the fifth year of simulation.

**Figure 8:**
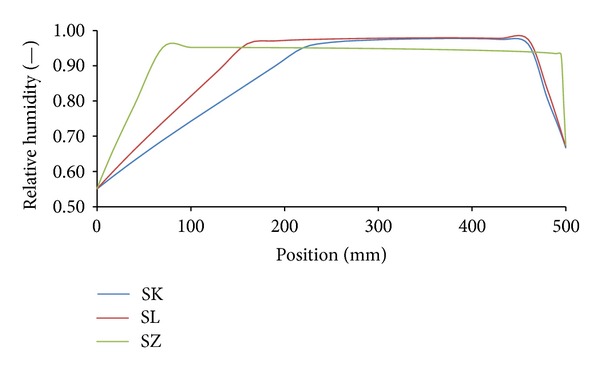
Relative humidity profiles, Šerák's climatic conditions, January 15, 3:00 a.m.

**Figure 9:**
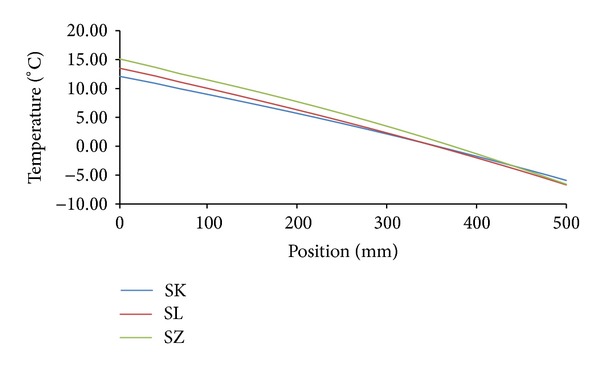
Temperature profiles, Šerák's climatic conditions, January 15, 3:00 a.m.

**Figure 10:**
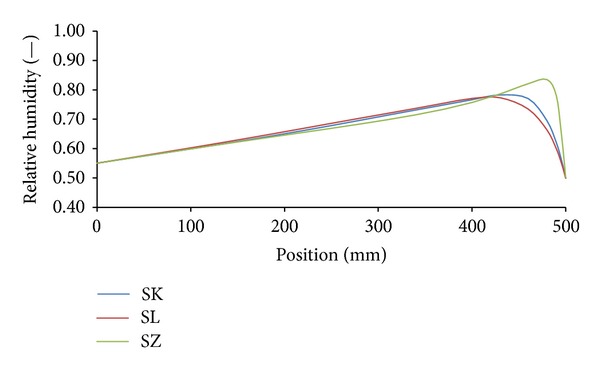
Relative humidity profiles, Holešov's climatic conditions, July 15, 3:00 p.m.

**Figure 11:**
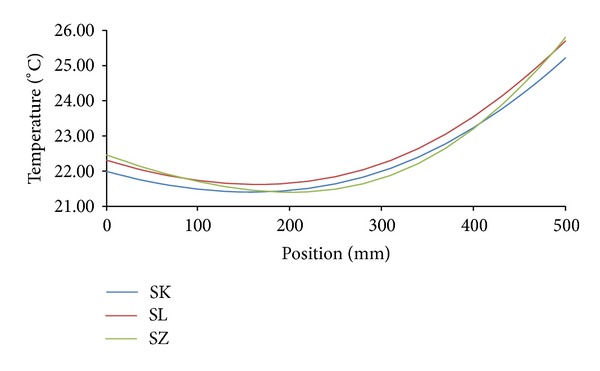
Temperature profiles, Holešov's climatic conditions, July 15, 3:00 p.m.

Temperature and moisture fields in the analyzed building envelopes were generated in a form of hourly nodal values of moisture content and temperature. These values were used for the construction of moisture or temperature profiles in the building envelope in any moment of the analyzed time period. Figures 8 and 9 show an example of relative humidity and temperature profiles for all investigated variations of building envelopes under Šerák's climatic conditions on January 15, 3:00 a.m, of a test reference year. Similarly, Figures 10 and 11 show the same profiles under Holešov's climatic conditions on July 15, 3:00 p.m. In all figures, the position 0 mm denotes the interior side of the envelope; the position 500 mm means the exterior side.

**Figure 12:**
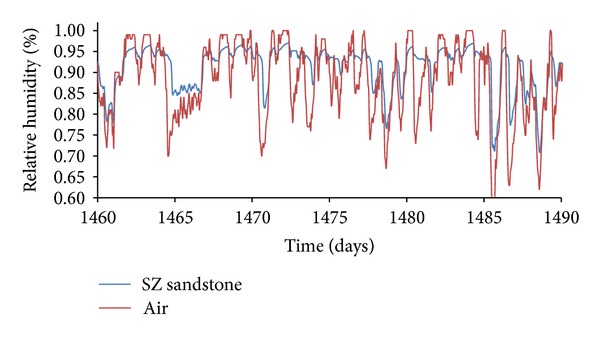
Relative humidity of air for SZ sandstone under Prague's climatic conditions, January.

The function of relative humidity of SZ sandstone in point 2 mm under the surface versus relative humidity of air during January under Prague's climatic conditions is captured in Figure 12.

It is obvious that temperature differences between different sandstones were not as significant as the differences in moisture content, represented by relative humidity. The highest temperature differences (3.01°C) were achieved on the interior side between SK and SZ sandstones under Šerák's climatic conditions, which corresponded to their different thermal parameters (Table 9). The assessment of the hygric performance of analyzed building envelopes appeared as a more sophisticated issue because several transport mechanisms were involved. It was essential to distinguish between the different types of moisture transport, that is, whether liquid moisture or only water vapor was transported. According to the results presented in Figures 8, 9, 10, and 11, among all the investigated materials SZ sandstone differed most significantly from the others. Comparing the moisture diffusivity functions presented in Figure 4 and water vapor sorption properties presented in Figure 7, SZ sandstone reached the highest values. In practice it meant that the SZ sandstone accumulated a higher amount of water vapor than other sandstones under the same conditions and thanks to the high values of moisture diffusivity it exhibited the fastest response to the changes in moisture conditions in the exterior.

### 3.3. Service Life Assessment

The number of freeze/thaw cycles appearing in the investigated sandstones was determined using a parallel evaluation of their hygric and thermal performance. One freeze/thaw cycle in the selected node of the finite-element mesh was counted only if the moisture content was higher than the maximum hygroscopic moisture content, as expressed by the corresponding value of relative humidity of 97.6%, and the temperature at the same time dropped below zero. These two conditions had to be met at least for six hours followed by at least six-hour lag, which was in compliance with the methodology for experimental determination of frost resistance of natural stones [18]. The freeze/thaw cycles appearance was detected in the node 2 mm under the external surface of building envelope. The freeze/thaw cycles counted in this point could cause material damage unlike the cycles counted on the surface of the envelope, and, as this point was very close to the exterior, the effects of weather conditions were still significant here.

In the presentation of obtained results, only representative graphs capturing moisture and temperature versus time functions are given, in order to illustrate the process of counting the freeze/thaw cycles in the three different sandstones subjected to the three different climatic conditions.

**Figure 13:**
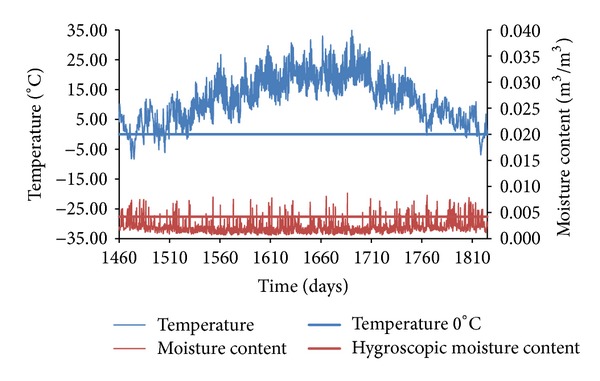
Temperature and moisture content versus time functions, SK sandstone, Prague.

Figure 13 shows moisture and temperature versus time functions of SK sandstone under Prague's climatic conditions. While temperature dropped below zero only during the winter period, moisture content reached the overhygroscopic range for short periods during the whole reference year. Therefore, there were only eight freeze/thaw cycles detected in SK sandstone envelope under Prague's climatic conditions.

**Figure 14:**
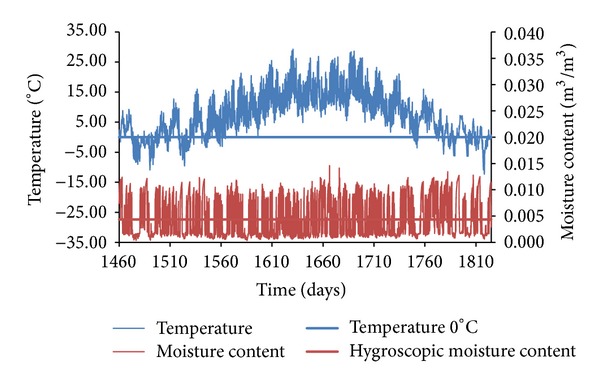
Temperature and moisture content versus time functions, SL sandstone, Šerák.

Completely different results were obtained when SL sandstone under Šerák's climatic conditions was assessed (Figure 14). Because of harsher weather conditions, the temperature of the building envelope 2 mm under the external surface was lower than in the previous case and the moisture content was higher, so that the presence of liquid moisture was more frequent. These circumstances created very good preconditions for the appearance of up to 33 freeze/thaw cycles per a reference year.

**Figure 15:**
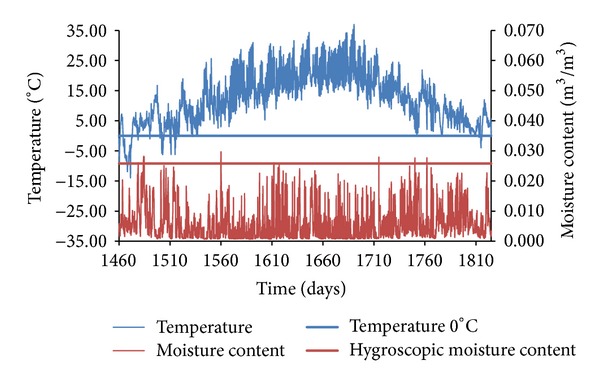
Temperature and moisture content versus time functions, SZ sandstone, Holešov.

The most convenient conditions for the service life of sandstone building envelopes from the point of view of their freeze/thaw resistance were observed in Holešov. Because of the relatively high temperatures and low relative humidity of air in the test reference year, not a single freeze/thaw cycle was counted. As it is presented in Figure 15 showing temperature and moisture content versus time functions of SZ sandstone, the moisture content reached the overhygroscopic range only several times per a reference year but temperature was always above zero at those times. That meant the conditions for the creation of a freeze/thaw cycle were not met.

Summary of the freeze/thaw cycles appearance for all investigated variations is presented in Table 10. The best results were achieved with the SZ sandstone, while the worst results were observed for SL. In a comparison with SK and SL, SZ sandstone had the lowest water vapor diffusion resistance factor (Tables 7 and 8). This led to a decrease of water vapor accumulation and its possible subsequent condensation. And even if liquid moisture appeared, thanks to the high values of moisture diffusivity (see Figure 6) it could be quickly transported and spread out. On the other hand, SK and SL sandstones released the contained moisture more slowly; thus it could be more easily accumulated and according to the sorption isotherms (see Figure 7) it could also be more easily condensed.

Combining the results of numerical simulation of freeze/thaw cycles appearance with the experimental measurements of freeze/thaw resistance, the assessment of service life was performed. The European standard [18] requires a value of frost resistance coefficient of natural stone higher or equal to 0.75. According to Mutlutürk et al. [30], there is an exponential development of rock properties exposed to cyclic freezing and thawing. Therefore, recounting the values presented in Table 6 according to logarithmic function, frost resistance coefficient value equal to 0.75 would be achieved approximately after 56 cycles for SK sandstone, after 213 cycles for SL sandstone, and after 77 cycles for SZ sandstone. It is important to notice that the evaluation of freeze/thaw cycles appearance was performed in the point located 2 mm under the external surface. It means that even if the limiting value of freeze/thaw cycles would be reached in this point, it does not mean exceeding the service life of the whole envelope, but only a damage of a thin surface layer accompanied by sandstone crumbling, cracks development, and so forth. In the opinion of the authors, only a disruption of more than 20–30 mm can be considered as a substantial damage of a natural stone-based building envelope due to the effects of freeze/thaw cycles.

The service life estimates of sandstone building envelopes are summarized in Table 11. One can notice that the service life of SL sandstone is higher than SK sandstone, despite its worse hygrothermal performance. This difference is caused by much better mechanical properties of SL sandstone, which makes it more resistant against weathering effects and freeze/thaw cycles in particular.

The obtained results of service life of sandstone building envelopes cover a relatively wide range of possibilities, because they take into account the differences both in the type of sandstone and in the dynamic climatic conditions applied on the exterior side of building envelope. On the other hand, they are specific concerning the type of rock in general and the climate is restricted to the conditions of Central Europe. Therefore, a comparison with the results presented by other research groups could be done in a limited extent only. In addition, only few researchers combined the experimental and numerical approaches to estimate the service life. They mostly focused just on the determination of the number of freeze/thaw cycles leading to material disruption [31] or changes in mechanical parameters [7–9]. Silva et al. [32, 33] investigated the service life of a large set of stone cladding samples, observing presence of visual surface degradation, loss of bound to the substrate, or loss of integrity as the main signs of service life exhausting. According to their results, the service life of studied stones lied between 38 and 232 years, depending on the type of stone, its color, distance from the sea, orientation and exposure to wind, rain, or damp. The service life presented in this paper was higher. However, unlike Silva et al. [32, 33] who basically just summarized the current state, observing real signs of damage on a selected set of stone claddings, this paper presented the service life prediction based on the results of experimental analysis. Therefore, the agreement can be considered as reasonable, taking into account all the different factors affecting the results reported in [32, 33] and in this paper.

Although the proposed method for service life assessment is very complex and is comprised of building materials research, experimental analysis of heat and moisture transport and storage parameters, and computational analysis using real dynamic weather data, several limitations still remain, which can affect the accuracy of results. The inaccuracies can be generated while service life is estimated by extrapolation of experimentally measured freeze/thaw resistance and calculated number of freeze/thaw cycles. As the material properties are gradually changed due to the effects of freeze/thaw cycles, it would be necessary, in order to obtain more precise results, to determine input material parameters not only as a function of moisture content or temperature but also as a function of freeze/cycles. Theoretically, it would be possible because the applied mathematical model allows including such material dependency. However, from the practical point of view, it would be very time consuming to measure all the material parameters in dependence on two or three independent parameters. Other inaccuracies can be generated by the fact that original building materials can be usually unavailable for experimental analysis (mostly destructive) because of heritage protection. Therefore, often only similar samples from other quarries can be obtained so that it is difficult to ensure that the material properties are really identical.

## 4. Conclusions

A noninvasive method for the service life assessment of building envelopes built of natural stones from the point of view of their freeze/thaw resistance was introduced in this paper. Within the frame of this study, three different types of sandstone were investigated under three different climatic conditions in the Czech Republic.

The presented method is based on a combination of experimental analysis and numerical simulation where the experimental measurement provides the material properties of the studied stones as the necessary input parameters for the numerical simulation of hygrothermal performance. The service life of building envelopes is then estimated using the results of hygrothermal simulations and the experimentally determined durability properties.

The results obtained in this paper indicated that hygric parameters of natural stones had the highest influence on the service life of the analyzed building envelopes. It was found that a high value of moisture diffusivity (>10^−6^ m^2^/s) of sandstone together with a low value of water vapor diffusion resistance factor substantially contributed to an improvement of freeze/thaw resistance. Therefore, the best performance was achieved for the SZ sandstone with the most favorable combination of hygric properties. On the other hand, SK sandstone provided the worst results.

The service life of the investigated sandstones was very variable and depended on both climatic conditions and the properties of stones. It lied between 29 and 399 years; for certain combinations of climate and stone it was even unlimited. These results underline the necessity to take the service life of natural materials with a great care and investigate it case by case.

## Figures and Tables

**Table 1:** Overview of studied sandstones.

Notation	SK	SL	SZ
Quarry location	Kocbeře	Libnava	Záměl

Age	Cretaceous (Cenomanian)		

Petrography	Glauconitic sandstone; massive texture; fine-grained	Glauconitic sandstone; banded texture; fine-grained	Glauconitic sandstone; schistose texture; medium-grained

Mineralogical composition	Quartz (83%); K-feldspar (2%); muscovite, rutile, tourmaline, zircon (1%)	Quartz (71%); K-feldspar (8%); glauconite (3%); zircon, titanite (1%)	Quartz (79%); glauconite (9%); feldspars (4%); muscovite (1%)

**Table 2:** XRF chemical composition of tested materials.

Substance	SiO_2_	Al_2_O_3_	Fe_2_O_3_	CaO	MgO	K_2_O	P_2_O_5_	*∑*
Amount [mass %]								
SK	97.45	1.870	0.368	0.035	0.071	0.090	—	99.887
SL	89.52	3.630	4.610	0.118	0.312	1.320	0.112	99.622
SZ	92.92	2.710	2.420	0.173	0.533	1.080	0.008	99.844

**Table 3:** Measurement accuracy of ISOMET 2114.

Measurement	Measurement range	Accuracy
Thermal conductivity	0.015–0.700 W/mK	5% of reading + 0.001 W/mK
	0.7–6.0 W/mK	10% of reading

Volumetric heat capacity	4.0*·*10^4^–4.0*·*10^6^ J/m^3^K	15% of reading + 1*·*10^3^ J/m^3^K

Temperature	−20–+70°C	1°C

**Table 4:** Basic physical properties.

Sandstone	Bulk density [kg/m^3^]	Matrix density [kg/m^3^]	Total open porosity [% m^3^/m^3^]
SK	2 227.7	2 653.5	16.1
SL	2 191.0	2 667.8	17.9
SZ	2 075.9	2 689.2	22.8

**Table 5:** Mechanical properties.

Sandstone	Compressive strength [MPa]	Young's modulus [GPa]
SK	52.9	17.4
SL	60.1	22.5
SZ	25.5	16.8

**Table 6:** Freeze/thaw resistance.

Sandstone	Freeze/thaw resistance (70 cycles) [—]
SK	0.70
SL	0.91
SZ	0.77

**Table 7:** Water vapor transport properties, dry cup method.

Sandstone	*δ* [s]	*D* [m^2^/s]	*μ* [—]
SK	1.44*E* − 11	1.95*E* − 06	12.7
SL	1.58*E* − 11	2.14*E* − 06	11.6
SZ	1.55*E* − 11	2.10*E* − 06	11.8

**Table 8:** Water vapor transport properties, wet cup method.

Sandstone	*δ* [s]	*D* [m^2^/s]	*μ* [—]
SK	2.46*E* − 11	3.39*E* − 06	7.4
SL	2.47*E* − 11	3.40*E* − 06	7.4
SZ	2.64*E* − 11	3.63*E* − 06	6.9

**Table 9:** Thermal parameters.

Sandstone		SK	SL	SZ
Thermal conductivity [W/mK]	Dry	3.53	2.71	2.10
	Saturated	5.21	4.64	3.88

Volumetric heat capacity [10^6^ J/m^3^K]	Dry	1.68	1.58	1.44
	Saturated	2.28	2.47	2.28

**Table 10:** Summary of freeze/thaw cycles appearance.

Location/type of sandstone	Freeze/thaw cycles		
	SK	SL	SZ
Prague	6	8	0
Šerák	29	33	7
Holešov	0	0	0

**Table 11:** Service life estimate of sandstone building envelopes.

Location/type of sandstone	Service life [years]		
	SK	SL	SZ
Prague	140	399	Unlimited
Šerák	29	98	165
Holešov	Unlimited	Unlimited	Unlimited
